# Design of the Jump Mechanism for a Biomimetic Robotic Frog

**DOI:** 10.3390/biomimetics7040142

**Published:** 2022-09-24

**Authors:** Jizhuang Fan, Qilong Du, Zhihui Dong, Jie Zhao, Tian Xu

**Affiliations:** State Key Laboratory of Robotics and System, Harbin Institute of Technology, Harbin 150001, China

**Keywords:** biomimetic robotic frog, jump robot, bioinspired structures

## Abstract

Frogs are vertebrate amphibians with both efficient swimming and jumping abilities due to their well-developed hind legs. They can jump over obstacles that are many or even tens of times their size on land. However, most of the current jumping mechanisms of biomimetic robotic frogs use simple four-bar linkage mechanisms, which has an unsatisfactory biomimetic effect on the appearance and movement characteristics of frogs. At the same time, multi-joint jumping robots with biomimetic characteristics are subject to high drive power requirements for jumping action. In this paper, a novel jumping mechanism of a biomimetic robotic frog is proposed. Firstly, the structural design of the forelimb and hindlimb of the frog is given, and the hindlimb of the robotic frog is optimized based on the design of a single-degree-of-freedom six-bar linkage. A simplified model is established to simulate the jumping motion. Secondly, a spring energy storage and trigger mechanism is designed, including incomplete gear, one-way bearing, torsion spring, and so on, to realize the complete jumping function of the robot, that is, elastic energy storage and regulation, elastic energy release, and rapid leg retraction. Thirdly, the experimental prototype of the biomimetic robotic frog is fabricated. Finally, the rationality and feasibility of the jumping mechanism are verified by a jumping experiment. This work provides a technical and theoretical basis for the design and development of a high-performance amphibious biomimetic robotic frog.

## 1. Introduction

After millions of years of evolution, organisms have developed dexterous movement mechanisms and agile movement patterns. Legged jumping robots transform the design of the robot from the biological form or structural function into prototypes in the engineering field through the principle of biomimetics [1]. For tasks with obstacle-crossing requirements, dangerous working environments, and low-gravity space environments, the jumping robot can give full play to its own mobile advantages and realize more flexible autonomous movement. Frogs combine excellent land jumping ability and flexible underwater swimming ability and have great research value. The complexity of their biological structure greatly increases the difficulty of designing frog-inspired robots. Therefore, the research on frog-inspired robots has been divided into research on frog-inspired jumping robots and research on frog-inspired swimming robots.

Due to the high energy density requirement of the jumping motion, most existing jumping robots use elastic elements combined with locking and releasing mechanisms to store and release elastic energy. Electric motors have advantages of size, price, ease of use, and control accuracy for robots with small loads. Most of the current frog-inspired jumping robots are designed to jump in this way. In 2008, Wang Meng from the Harbin Institute of Technology designed a simplified single-degree-of-freedom rhombic-shaped four-bar biomimetic robotic frog hindlimb mechanism [2], which uses the motor to pull the rope to store spring energy. In 2008, Li Tao from the North China University of Technology designed a frog-inspired jumping robot using a spring, dial linkage, and wire rope structure to complete the elastic energy storage and jumping [3]. The frog jumping robot designed by CSIR in India in 2011 combines spool winding and ratchet release mechanism with a four-link spring mechanism on the main trunk [4]. In 2013, the Korea Advanced Institute of Science and Technology adopted a four-link structure to imitate the hind legs of a frog, used a low-power motor as a torsional drive to store the elastic potential energy on the rubber, and proposed a 22.5 g frog-inspired small jumping robot. It can jump 2.5 m high (58 times its body length) [5]. In 2014, the Japanese public Hakodate Mirai University proposed a double-joint type leg mechanism [6]. The design uses two types of actuators to achieve instantaneous jumping and quadruped walking. One is a cam spring mechanism that is compressed and released by a motor, which pulls the tendons running through each joint to achieve instantaneous extension of one leg. The other is a servo motor for precise control of each joint. In 2016, Zhang Qinran developed a C-type flexible reed to replace the calf, ankle, and fins and designed a cam compression spring mechanism to store and release elastic potential energy [7]. In 2016, Lu Chenjun designed a prototype of a frog-inspired jumping robot. This robot used a parallel four-bar mechanism and a synchronous belt mechanism combined with a one-way bearing to realize the storage and release of spring elastic potential energy [8]. In 2017, Yan Jia proposed a frog-inspired variant mobile robot design scheme that can realize the conversion of land jumping mode and water breaststroke model. The motor pulls the spring through the crank slider mechanism, and the elastic energy is instantaneously released through the rocker slider trigger [9]. In 2020, Hong Chong et al. proposed jumping strategies based on series-elastic actuators (SEA) without latch mechanisms, combining SEA with a parallel elastic four-link structure, and designed a frog-inspired jumping robot mechanism. The robot uses a motor to store elastic potential energy and automatically trigger the release. The prototype weighs 100.7 g and can jump 1.3 m high [10]. This type of driving method requires the design of a gear reducer, elastic element, and energy release mechanism. Only by selecting appropriate elastic elements and optimizing robot kinematics, dynamics, and motor control can the robot achieve the desired results [11].

Hydraulic linear actuators have a higher power-to-weight ratio than electric motors, such as the Big Dog robot developed by Boston Dynamics [12]. In 2017, Li Yang designed a frog-inspired jumping robot based on a hydraulic drive [13]. The overall leg mechanism is still similar to Wang Meng’s diamond-shaped four-bar spring mechanism. However, the speed response of the hydraulic linear actuator is nonlinear, requiring a fluid compressor or an external energy storage device, which is inconvenient to be onboard. In order to be more in line with the principle of biomimetics, some research institutions have begun to use pneumatic muscles as actuators, such as the frog-inspired robot “Mowgli” [14] and the frog-inspired robot developed by the Harbin Institute of Technology [15,16]. Compared with hydraulic linear actuators, pneumatic muscles have many properties of natural muscles. However, pneumatic muscles also require air compressors or external high-pressure air sources for energy supply. The overall structure of this kind of robot is large, and the control system is complex. The biomimetic frog jumping robots in [14,15,16] also only use an external air source for energy, and the weight of the whole prototype is more than 5 kg. Therefore, the pneumatic and hydraulic drive is suitable for large and heavy-load biomimetic jumping robots but not for small robots or robots with high jump height/weight ratio requirements. As an excellent deformable material, shape memory alloy (SMA) has a good power-to-weight ratio and can also provide enough energy to help robots jump. The frog-inspired jumping mechanism in [17] is realized by an intelligent composite microstructure and SMA spring actuator, which replaces traditional actuators, reducers, and elastic elements to reduce the overall size and weight, and can jump five times its own length. However, its driving force is too small; it is a miniature jumping mechanism that jumps only by an external power cord. There are also many jumping robots driven by explosive soft actuators [18,19,20,21]. From 2019 to 2021, the Harbin Institute of Technology successively developed two frog-inspired jumping robots using explosive-driven soft actuators, with a jumping distance of 1.4 m and a jumping height of 0.6 m [22,23]. The chemical fuel drive does have the advantage of high energy density, but the continuous supply of fuel is not as convenient as the motor. In addition, the characteristics of easy leakage, flammability, and explosion may bring certain dangers to robotic experiments.

The geometry and size of the currently designed frog-inspired jumping robots are obviously different from that of natural frogs. Most of the jumping mechanisms use a simple four-bar linkage mechanism, which has a poor bionic effect on the appearance and movement characteristics of frogs. At the same time, the multi-joint jumping robot with bionic characteristics has high driving power requirements for the actuator due to the jumping action. In this paper, a novel jumping mechanism of a biomimetic robotic frog is proposed, which can well achieve the geometric shape and joint motion range similar to natural frogs, with a longer stroke ratio and better bionic effect. Moreover, the compact elastic energy storage and trigger mechanism is used to realize the complete jumping process of the robot, that is, elastic energy storage and regulation, energy release, and rapid leg retraction. The mechanism addresses the instantaneous high-power drive demand of the actuator for jumping motions.

The rest of this article is organized as follows. Section 2 introduces the design of the jumping mechanism, including the design of the forelimb and hindlimb, the jumping simulation, and the design of the elastic energy storage and trigger mechanism. Section 3 gives the prototype fabrication, jumping experiments, and discussion of the experimental results. Section 4 concludes this article.

## 2. Design of the Jump Mechanism

### 2.1. Design of Forelimb and Hindlimb

According to the research on the biological characteristics of frogs in the early stage of our research group [2,16], the mass of the frog is mainly concentrated in the body, and the mass of the limbs accounts for a small proportion of the total mass. The forelimbs are short and mainly play a role in supporting the front of the body. The hindlimbs are slender, providing propulsion for jumping and swimming. The length of the thigh and calf of the frog hindlimb is basically the same. The length of the upper and lower arms of the forelimb is also basically the same. The total length of the forelimb is about half of the total length of the hindlimb. In the design of the forelimb and hindlimb of the biomimetic robotic frog, we also carry out the size design according to this proportional relationship.

The hip joint of the frog’s hindlimb has 3 degrees of freedom (flexion and extension, abduction and adduction, and internal and external rotation). The knee joint, ankle joint, and tarsometatarsal joint each have 1 degree of freedom (flexion and extension). The forelimb’s shoulder joint has 3 degrees of freedom, and the elbow and wrist joints have 1 degree of freedom each. The angle of each joint during the actual take-off stage of the frog; the flexion and extension of the hip joint is 135°, the abduction and adduction movement is 40°, the internal and external rotation movement is first reduced by 10° and increased by 40°, the knee joint flexion and extension movement is 155°, and the ankle joint flexion and extension movement is 150°. The flexion and extension movement of the plantar joint first increased to 38° and then decreased to −30°. The size parameters of the forelimb and hindlimb of the robot are optimized by taking the change of each joint angle as the constraint.

**Figure 1:**
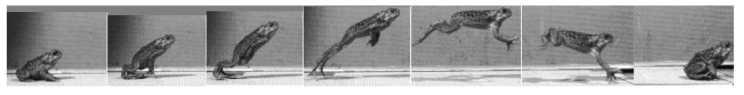
The frog jumping process [13].

Based on the postures and movements of the frog’s body and limbs during jumping, the jumping process can be divided into three stages (Figure 1).

1. The take-off stage. During the take-off phase, the frog begins to move from a crouched state until its foot leaves the ground. In this stage, the muscles of the hindlimbs are stimulated to contract rapidly, causing the hindlimbs to unfold instantly. The forelimbs support the body to adjust the take-off angle. After leaving the ground, the forelimbs retract to both sides of the torso. During this period, the hip, knee, and ankle joints start to move. When the flippers completely leave the ground, the take-off stage ends.
2. The flight stage. In this stage, the frog moves from the flipper off the ground to the forelimbs touching the ground again. The hindlimbs are gradually retracted from the fully extended state, and the forelimbs are gradually stretched forward from both sides of the body. This action increases the moment of inertia so that the frog maintains the balance of its body posture in the air. When the forelimbs touch the ground again, the flight phase ends.
3. The landing stage. The forelimbs touch the ground, and the shoulder, elbow, and wrist joints move in coordination, gradually lowering the frog’s center of gravity. After the hindlimbs are fully retracted and touch the ground, the center of gravity shifts back, and the frog stabilizes again in preparation for the next jump.

The muscle force of frogs remains constant throughout the contraction to ensure maximum work capacity [24]. The peak energy released in the late stage of take-off is caused by elastic energy storage elements such as muscles or tendons [25]. Some studies have shown that series-elastic elements can be used to amplify the peak power output of tendon units during jumping [26,27,28], which is essential for achieving high-performance jumps. The jumping process of frogs requires extremely high power density. It is difficult to simulate the effect of muscle and achieve high power output by using a motor to control joints directly. Therefore, we still need to use energy storage elements. The spring drive is one of the most widely used driving methods. One way is to use springs to achieve energy storage and rapid release so that the robot can take off quickly. The size range of the springs varies widely, which can drive jumping robots of different sizes.

By observing the jumping movement and swimming movement of frogs, it is found that frogs mainly generate thrust for jumping and swimming through developed hindlimbs. For frogs, hindlimbs are more important than other parts because they are the source of most of the thrust. Therefore, the design of hindlimbs and transmission mechanisms is crucial for the development of high-performance biomimetic robotic frogs. The hindlimbs of the frog have seven degrees of freedom and are attached to a flexible webbed foot to generate thrust. It is difficult to combine all the degrees of freedom into a single electromechanical system. Although the hip joint of frogs has three degrees of freedom, the flexion and extension of the hip joint play a major role during the jumping process. The ankle joint moves almost in a straight line with respect to the hip joint. By coupling the joints as much as possible to minimize the total effective degrees of freedom, we design a frog-inspired limb structure to imitate the movement of the hindlimbs, thigh, and calf. Furthermore, the actuator is placed on the main torso to reduce limb weight and avoid high inertia effects. By integrating the limb action characteristics of frog jumping movement, the limb mechanism also needs to realize the approximate linear movement of the endpoint so as to complete the jumping action.

It is difficult for single-degree-of-freedom four-bar linkages that generate linear motion to achieve the biological size ratio and motion range of the biological frog limbs. These kinds of mechanisms are either shorter than the stroke or have a poor bionic effect, whose free design parameters are less. Bioinspired open-loop and closed-loop multi-link structures can not only imitate the jumping mechanism of animals but also can be designed to be similar to biomimetic objects in natural geometry and size [29]. The single-degree-of-freedom planar six-bar mechanism has the potential to follow an approximate linear trajectory after the size optimization design [30]. Based on the Stephenson six-bar mechanism, we carry out the optimal design of the mechanism parameters. Aiming at the biological structural characteristics and action characteristics of the frog hindlimbs, we set the following conditions for the design of the biomimetic frog limb structure:

(1) The size of the connecting rods corresponding to the thigh and the calf is close to that of the hindlimbs of the frog;
(2) The rotation angle of the connecting linkage is close to that of the hip joint and knee joint of the frog;
(3) The movement process of the linkage mechanism is similar to the hindlimb extension process of the frog jumping process;
(4) The end of the link mechanism corresponding to the ankle joint outputs the expected linear trajectory;
(5) The other pivot positions of the link mechanism are compact, and the overall shape and geometry of the mechanism are similar to that of the frog’s hindlimb.

**Figure 2:**
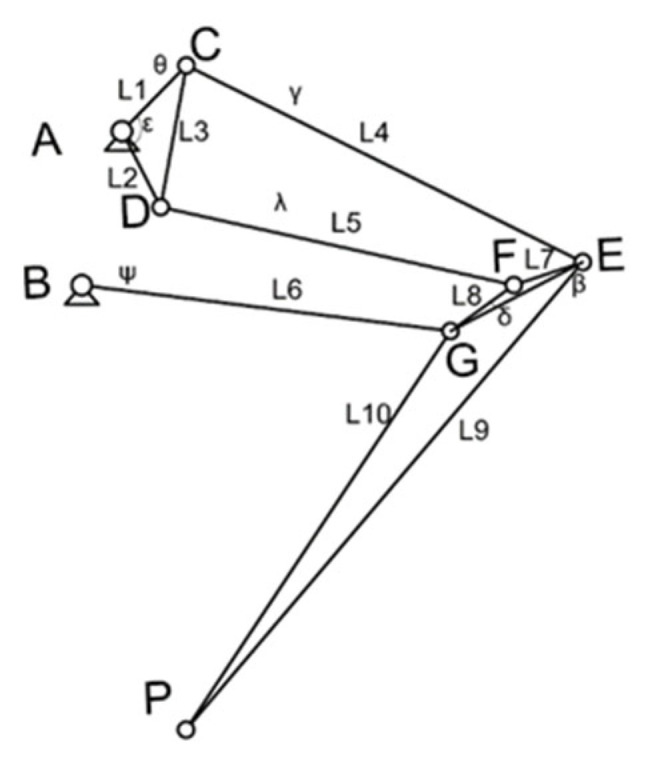
The modeling of the hindlimb linkage mechanism.

The modeling of the hindlimb linkage mechanism is shown in Figure 2. The fixed pivots A and B are fixed on the torso of the robot. The BG linkage can be regarded as the thigh, and the GP linkage can be regarded as the calf. In order to make the mechanism maintain a single degree of freedom on the expected output linear trajectory, other pivots and links serve as auxiliary mechanisms.

Aiming at the endpoint trajectory approximating a straight line, the comprehensive equation of the mechanism loop should be established first. The dimensional parameters of the mechanism should be obtained by optimization function based on the trajectory points. The fixed pivots A and B, the active pivots C, D, E, F, G, and the end trajectory tracking point P are modeled by plane kinematics. Given the initial coordinates $\left(A_{x},A_{y}\right)$ of the fixed pivot A, the initial position vector of the pivot A can be expressed as:

(1) $$A_{0}=A_{x}+A_{y}i.$$

where i is the imaginary unit.

Fixed pivot B is represented as:

(2) $$B_{0}=B_{x}+B_{y}i.$$

Similarly, other pivots C, D, E, F, G, and P can be represented by complex numbers.

$L_{i}(i=1,2\cdots \cdots 10)$ is a complex variable, set

(3) $$\begin{array}{l} \vec{\mathrm{AC}}{=L}_{1},\vec{\mathrm{AD}}{=L}_{2},\vec{\mathrm{CD}}{=L}_{3},\vec{\mathrm{CE}}{=L}_{4},\vec{\mathrm{DF}}{=L}_{5},\\ \vec{\mathrm{BG}}{=L}_{6},\vec{\mathrm{EF}}{=L}_{7},\vec{\mathrm{FG}}{=L}_{8},\vec{\mathrm{EP}}{=L}_{9},\vec{\mathrm{GP}}{=L}_{10}. \end{array}$$

The rotation angles corresponding to the five motion links ACD, CE, DF, BG, and EFGP shown in Figure 2 are θ,γ,λ,ψ,β, respectively. $A,B,C,D,E,F,G,P_{0}$ represent the initial position. $A,B,C_{j},D_{j},E_{j},F_{j},G_{j},P_{j}$ represent the movement position of the mechanism. $\theta_{j},\gamma_{j},\lambda_{j},\psi_{j},\beta_{j}$ represent the rotation angle of the corresponding moving link relative to the initial position when the mechanism moves to the jth position, expressed by the following exponential rotation operator:

(4) $$Q_{j}=e^{i\theta_{j}},R_{j}=e^{i\gamma_{j}},S_{j}=e^{i\lambda_{j}},T_{j}=e^{i\psi_{j}},U_{j}=e^{i\beta_{j}}\;.$$

Three closed loop vector equations are obtained from the fixed pivot to the endpoint:

(5) $$\left.\begin{array}{l} A+Q_{j}(C-A)+R_{j}(E-C)+U_{j}(P_{0}-E)=P_{j}\\ A+Q_{j}(D-A)+S_{j}(F-D)+U_{j}(P_{0}-F)=P_{j}\\ B+T_{j}(G-B)+U_{j}(P_{0}-G)=P_{j} \end{array}\right\}\;.$$

The conjugate complex form is obtained from the above closed loop vector equation:

(6) $$\left.\begin{array}{l} \bar{A}+\bar{Q_{j}}(\bar{C}-\bar{A})+\bar{R_{j}}(\bar{E}-\bar{C})+\bar{U_{j}}(\bar{P0}-\bar{E})=\bar{P_{j}}\\ \bar{A}+\bar{Q_{j}}(\bar{D}-\bar{A})+\bar{S_{j}}(\bar{F}-\bar{D})+\bar{U_{j}}(\bar{P0}-\bar{F})=\bar{P_{j}}\\ \bar{B}+\bar{T_{j}}(\bar{G}-\bar{B})+\bar{U_{j}}(\bar{P0}-\bar{G})=\bar{P_{j}} \end{array}\right\}\;.$$

The geometric constraint function of the connecting linkage model is obtained by solving the closed loop equation. Other constraint functions are set according to the above constraints. The specific values are adjusted according to the optimization results. The core idea is to achieve trajectory optimization by solving the optimization variables to make the difference between a series of actual toe position points and expected toe position points as small as possible. Construct the objective function as follows:

(7) $$\min f=\sum_{j}^{N}\left(P_{j}-P_{jpre})(\bar{P_{j}-P_{jpre}}\right)\;.$$

According to the geometry and size ratio of the hindlimb of the frog and the design requirements of the biomimetic robot, the lengths of the thighs and calves of the mechanism are similar, and the rotation angles of the hip and knee joints are equivalent to the extension of the hindlimb of the frog. Setting the initial size BG=100 mm,GP=105 mm, the optimization variables are the initial position coordinates of each pivot. Equations (5) and (6) are equality constraints. Some inequality constraints are set according to the actual pivot assembly. The interior point method is set by MATLAB’s built-in function fmincon for programming calculation. The results of the optimal design of the linkage length are shown in Table 1.

**Figure 3:**
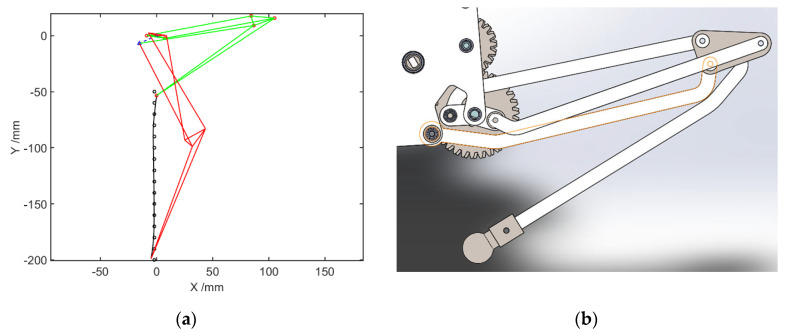
Hindlimb optimization mechanism: (**a**) The optimization results of the link; (**b**) Structural model of hindlimb mechanism.

The optimization result is shown in Figure 3a, in which the green is the initial position of the linkage, the red is the final position of the linkage, and the black solid line is the end motion trajectory. In the optimization design of the linkage length of the mechanism based on the interior point method in this paper, the standard deviation of the endpoint trajectory is 0.7854. The optimization result approximates the expected trajectory. According to the optimization results, a three-dimensional structural model of the hindlimb mechanism is designed, as shown in Figure 3b.

The frog’s forelimbs are relatively slender and short, which is not the main source of jumping power. Its main functions are body support, take-off posture and direction adjustment, adjustment of body balance, and landing buffer. Therefore, the forelimbs of the robot also need to have the following three basic functions:

(1) Support the robot;
(2) Adjust the overall pitch angle of the robot;
(3) Extend the front limbs when swinging forward to make the robot land smoothly.

**Figure 4:**
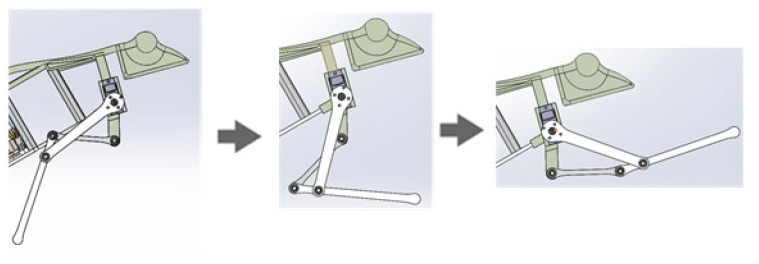
Forelimb Structural Model.

Therefore, the forelimb design can be simplified into a planar four-link mechanism with only shoulder joints and elbow joints (Figure 4), driven by a single steering gear. In the initial position, the distance between the wrist and the shoulder is minimal when the forelimb is in the middle of the stroke. When the front limb swings back, the distance between the wrist and the shoulder increases, which can be used to adjust the body pitch angle when the robot takes off. When the front limb swings forward, the distance between the wrist and the shoulder also increases, which can be used for aerial posture adjustment and preparation for landing.

### 2.2. Kinematics Simulation of Mechanism Simplified Model

With the help of simulation software, the feasibility of the robot model for the forelimb and hindlimbs designed in the upper section is verified to ensure the correctness of the research plan. Firstly, a simplified model of the robot with front and rear limbs is established. Then, the ADAMS simulation software is used to carry out a kinematic simulation analysis of the feasibility of the jumping motion of the robot’s mechanical structure.

Using Solidworks software, a simplified forelimb, hindlimb, and frame structure model of the robot is established and imported into ADAMS. The material properties of each part are modified. Since the weight of the motor, battery, and other parts with a large weight is attached to the frame, the weight of the frame is set to 400 g. In this simulation, the actual motor drive is replaced by directly specifying the motion of the active linkages. In addition to the default gravity, the contact force for the robot’s sole and floor and forelimb end linkage and floor were set. Normal force was selected as collision. Stiffness was set to 1.0 × 10^5^. The force index was set to 2.2. Damping was set to 10.0. The penetration depth was set to 0.1. In addition, the setting of the spring force was performed according to the properties of the torsion spring that was initially selected. The stiffness coefficient of the left torsion spring was set to 5 Nmm/deg. The damping coefficient was set to 2.79 × 10^−3^ Nmms/deg, and the rotation angle was set to 180° during operation. Since the left and right sides of the hind limb are in a mirror image relationship, the stiffness coefficient of the other torsion spring is −5 Nmm/deg, and the other parameters remain the same.

**Figure 5:**
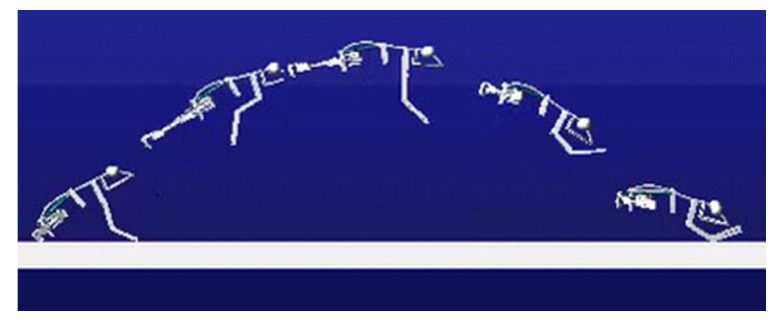
Simulation process of jumping motion of simplified frog mechanism model.

**Figure 6:**
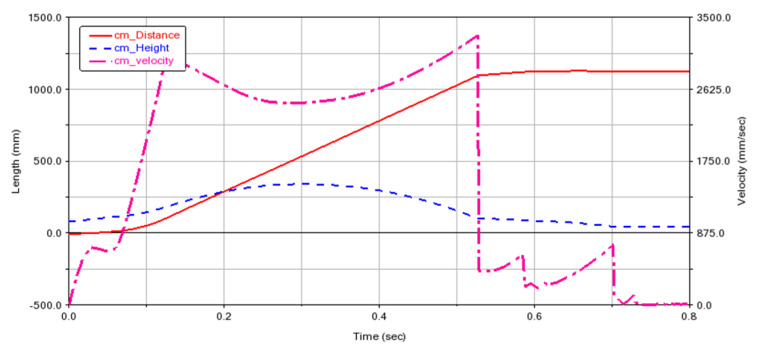
Simulation results of jumping motion of simplified frog mechanism model.

**Figure 7:**
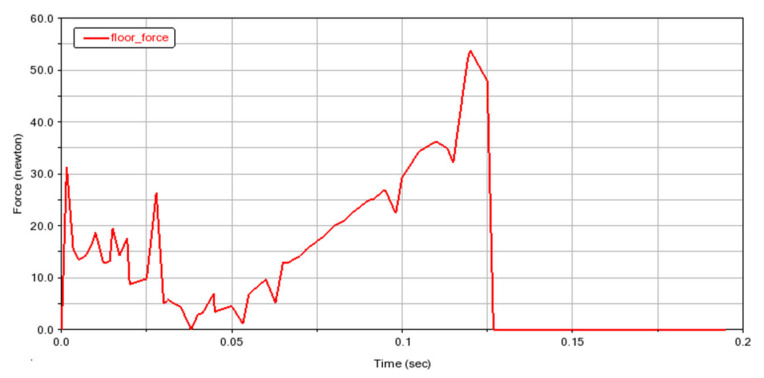
The ground reaction force of the ankle in simulation.

The simulation process is shown in Figure 5. The simulation results are shown in Figure 6. The jump distance is 1.1 m, and the jump height is 0.3 m. In Figure 6, the velocity refers to the vector sum of horizontal velocity and vertical velocity of the robot’s center of mass. The critical point (T = 0.3 s) is the jump to the highest point, after which the gravitational potential energy is again converted to kinetic energy. Therefore, the velocity increases. Both the simulation and experiment show the feasibility of using this mechanism to achieve biomimetic frog jumping. The change in the ground reaction force is shown in Figure 7. It was found that the ground reaction force is not gradually reduced by the torsion spring force but is related to the output efficiency of the hindlimb transmission mechanism.

### 2.3. Elastic Energy Storage and Trigger Mechanism

In order to meet the high energy density characteristics of robot jumping, the energy storage and trigger mechanism of the robot are designed. The energy storage drive mechanism of the hind limbs needs to meet the following functional requirements:

- Energy regulation and storage of different elastic potential energy when accumulating;
- Rapid release of elastic potential energy when jumping with outstretched legs;
- Quick response to the leg retraction after jumping.

**Figure 8:**
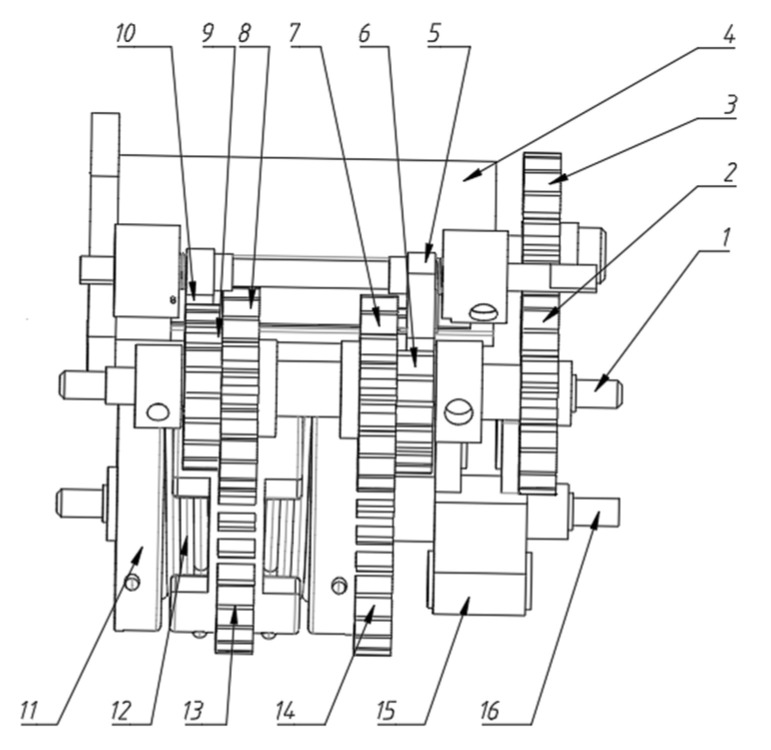
Assembly drawing of energy storage transmission mechanism.

**Figure 9:**
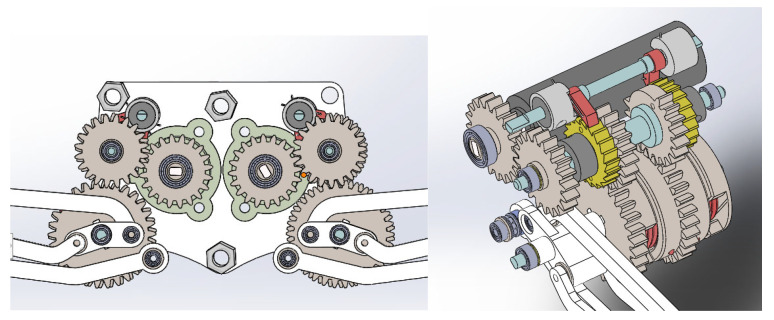
3D structural model of transmission structure.

In order to meet the above requirements, the elastic energy storage and trigger mechanism are designed, and two-stage gear transmission is adopted. The mechanism mainly includes one-way bearings, ratchets, pawls, incomplete gears and torsion springs, etc. The assembly diagram of the energy storage transmission mechanism is shown in Figure 8, and the transmission structure model is shown in Figure 9. The extension and contraction of the hindlimb link mechanism and the storage and release of the energy of the torsion spring are controlled by the forward and reverse rotation of the motor. The overall structure is described in conjunction with the assembly drawing.

The power source, 4, is a steering gear. The transmission components, 2 and 3, are driven by a gear pair. Gear 2 is fixed on shaft 1 and rotates together.

The first incomplete gear, 7, and the second incomplete gear, 8, are, respectively, fixed with the first ratchet, 6, and the second ratchet, 9. The inner rings of the first incomplete gear 7 and the second incomplete gear 8 are, respectively, sleeved with one-way bearings. Then, two one-way bearings are assembled on shaft 1 in opposite directions.

The first incomplete gear 7 meshes with the first gear 14 for realizing the extension and contraction of the leg. The first gear 14 is fixedly connected with the output link 15 of the hip joint.

The hip joint output link 15 is fixedly connected with the output shaft 16. The output shaft 16 is fixedly connected with the torsion spring fixing wheel 11 for installing the torsion spring 12 on the side of gear 13.

The second incomplete gear 8 meshes with the second gear 13 to control the torque of the torsion spring. The energy stored in the torsion spring is adjusted by rotating the second gear 13. The inner ring of the second gear 13 is sleeved on the output shaft 16 through a bearing.

**Figure 10:**
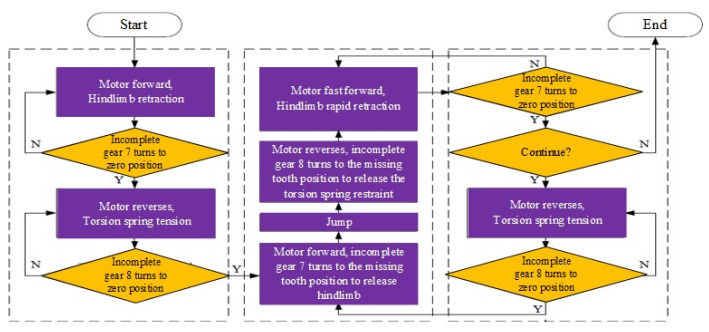
Jump control process of elastic energy storage trigger mechanism.

The jumping of the robot legs is realized by the time-sharing control drive mechanism of a single motor. Figure 10 shows the control process of one jumping cycle. The specific implementation is as follows.

(1) Take off stage. The steering gear 4 rotates forward. The first incomplete gear 7 is driven to rotate through the gear pairs 2 and 3. The first incomplete gear 7 meshes with the first gear 14. The gear turns to the first two teeth of the toothless area, which represents zero position. When the first incomplete gear 7 reaches the zero position from meshing, the first gear 14 drives the hip joint output link 15 to rotate so as to realize the contraction of the hind limbs. During this process, under the action of the one-way bearing, the second ratchet wheel 9 and the second pawl 10, the second incomplete gear 8 does not rotate. The output shaft 16 is relatively opposite to the second incomplete gear 8 and the second gear 13 rotates.
(2) After that, the steering gear 4 is reversed. The second incomplete gear 8 is driven to rotate in the reverse direction through the gear pair 23. The second incomplete gear 8 meshes with the second gear 13. When the second incomplete gear 8 reaches the zero position from meshing, the torsion spring 12 twists and stores elastic potential energy. Similarly, in this process, under the action of the one-way bearing, the first ratchet wheel 6 and the first pawl 5, the first incomplete gear 7 does not rotate. Only the second gear 13 rotates. The spring rotates relative to the output shaft 16.
(3) Then, the steering gear 4 rotates forward. The first incomplete gear 7 rotates. The first incomplete gear 7 and the first gear 14 are no longer meshed. At this time, the first gear 14 is not constrained by the meshing force. Under the action of the elastic potential energy stored by the torsion spring 12, the hind limbs are rapidly extended to realize jumping.
(4) In the flying stage, when the machine legs leave the ground, they need to be retracted quickly. The motor is in a high-speed state and cannot provide large torque. Therefore, it is necessary to release the restraint of the torsion spring gear end first. The steering gear 4 is reversed. The second incomplete gear 8 rotates in the reverse direction. The second incomplete gear 8 and the second gear 13 are no longer meshed. Then the second gear 13 no longer compresses the torsion spring 12 so that the torsion spring 12 can be restrained. Then the steering gear 4 rotates forward again. The first incomplete gear 7 rotates until it meshes with the first gear 14 so that the hind limbs can be quickly recovered without the restraint of the spring force. At this point, a cycle of the extension and contraction of the leg is completed. The above process is repeated to achieve continuous jumping.

## 3. Prototype and Experiment

### 3.1. Prototype Fabrication

The energy stored in the torsion spring is:

(8) $$E=\frac{1}{2}k\varphi^{2}.\;$$

where E is the energy stored by the torsion spring, k is stiffness coefficient of torsion spring, φ is rotation angle of torsion spring.

The spring energy is converted into kinetic energy,

(9) $$\frac{1}{2}k\varphi^{2}=\frac{1}{2}mv^{2}.$$

According to the oblique throwing motion formula, the pitch angle between the take-off and the horizontal ground is set as θ, horizontal jump distance is set as L,

(10) $$\begin{array}{l} v*\sin\theta =gt\\ L=2tv*\cos\theta \end{array}$$

According to the above equation,

(11) $$k=\frac{mgL}{2\varphi^{2}\sin\theta \cos\theta }.$$

Combined with the jump simulation analysis in Section 2.2 and the above formula, and referring to the size parameters such as the number of coils of the torsion spring, wire diameter, middle diameter, and weight, a torsion spring with a stiffness coefficient of 5 Nmm/deg was selected. Two torsion springs with a wire diameter of 1.5 mm, a middle diameter of 20.5 mm, and a number of turns of 5 are used in parallel on both sides of the gear, thereby reducing the deformation of the mechanism caused by the torsion of the torsion spring. Taking into account factors such as the energy conversion rate, torsion spring damping, transmission mechanism friction, the inability of the legs to move in complete consistency, and the air resistance during jumping, the actual jumping distance will be slightly reduced.

The motor needs to drive the torsion spring to reach the maximum torsion angle of 180°. The reduction ratio between the motor output shaft and the torsion spring is 1.5. Therefore, the maximum torque of the motor should be greater than:

(12) $$T_{\max}=\frac{k*180}{1.5}=0.6\;\mathrm{Nm}.$$

The motor needs to drive the gear set to complete the leg retracting action when the robot is in the air. The air time is set to 0.25 s. When retracting the legs, gear 14 needs to rotate 180°. The reduction ratio to the motor is 1.2, and the motor speed requirements are:

(13) $$n=\frac{180}{1.2*0.25}*\frac{60}{360}=100\;\mathrm{RPM}.$$

That is, the no-load speed of the motor should be greater than 100 RPM. Therefore, the DS_BN22 servo can meet the requirements, and its main performance is shown in Table 2.

Since the robot’s forelimbs do not provide power for take-off, the selection of the forelimb motors mainly focuses on the rotation speed, motor self-weight, and control stability. Considering the small overall size of the robot, micro servo servos can be used for driving.

Rotation speed requirements: assuming that the time lag time of the robot is 0.15 s for short-distance jumping (longer time for long-distance jumping), the rotation speed of the forelimb motor needs to be able to rotate from the backward support posture to the forward straight landing posture during this time period. According to the structure size, the motor angle between the two attitudes is 90°. Then the requirements for the motor rotation speed are:

(14) $$n=\frac{90}{0.15}*\frac{60}{360}=100\;\mathrm{RPM}.$$

That is, the no-load speed of the motor should be greater than 100 RPM. The SCS0009 micro servo steering gear is selected as the motor for driving the front limbs. Its no-load speed is 143 RPM, which meets the speed requirements and has a small self-weight. The internal integrated potentiometer realizes a closed-loop position, which can perform more accurate position control. The main performance parameters of SCS0009 are shown in Table 3.

In the processing and preparation of the robot body, different properties of materials need to adapt to different requirements according to different structures and functions. The explosiveness of the locomotion of jumping robots requires light and strong materials for the robot body [31] so that the robot can jump higher and farther and also can be strong enough to avoid damage on landing. The performance parameters of commonly used lightweight and high-strength materials are shown in Table 4.

Due to the low weight and low surface friction coefficient of the material properties, POM was chosen for the cams, gears, and joints. Carbon fiber is anisotropic. When the direction of use is correct, the carbon fiber material has higher strength and lower density. Carbon fiber is used as the limb skeleton. The main body of the robot is 3D printed with nylon material to minimize the weight-to-strength ratio. The processing of lightweight materials can control the weight of the robot to less than 500 g.

**Figure 11:**
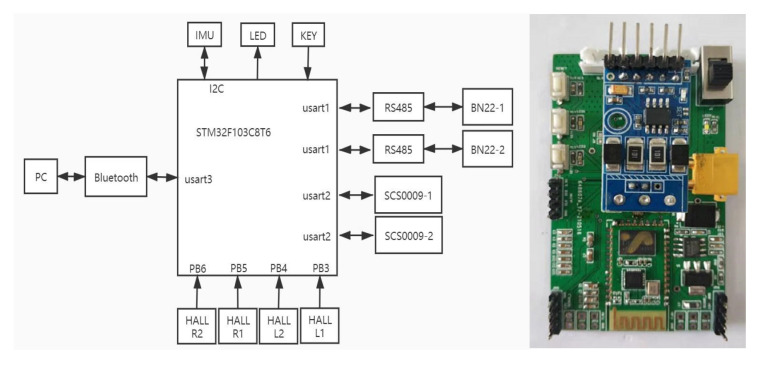
Hardware circuit block diagram and circuit board.

Based on the STM32F103C8T6 32-bit microcontroller, the hardware circuit of the robot is built. The peripheral circuit is designed on the basis of the minimum system, including the wireless communication of the module, the reading of the fuselage attitude data of the MPU9250 gyro sensor, and various basic interfaces and status indications, etc. The block diagram of the hardware circuit structure is shown in Figure 11.

**Figure 12:**
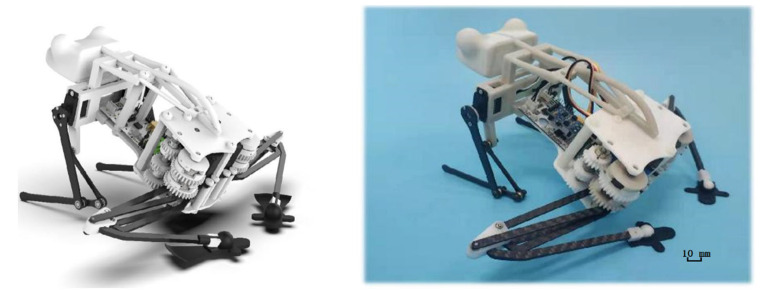
The overall structure model of the robot and the actual prototype.

Based on the above design, it adopts an overall shape similar to a frog, consisting of three parts: hind limbs, forelimbs, and body. Each part is designed according to the function of the corresponding part of the frog. The hind limbs mainly provide the force and energy for jumping; the forelimbs assist the ground when taking off, adjust the take-off angle, and buffer when landing; the body connects the limbs and installs the electric motor, circuit, and power supply. In order to reduce the mass of the limb structure as much as possible to improve the efficiency of jumping, all the driving elements of the limb joints are placed in the main torso. The torsion spring is driven by the motor gear to realize the adjustment of the hindlimb joints. The overall structure model of the robot and the actual prototype are shown in Figure 12. The overall size of the robot is 210 mm long, 125 mm wide, and 60 mm high.

### 3.2. Experiments and Results

The host computer program is developed using C# to control the frog jumping robot. The user interface is mainly divided into a serial port setting module, a demonstration module, and a debugging module. The lower computer program is written in the KEIL MDK 5 development environment to control and use each motor and sensor module, collect data, and process data. Each action of the robot is indicated and controlled by the corresponding FLAG action flag variable, and the corresponding Bluetooth serial communication protocol is configured. In this way, various test experiments are carried out on the actual motion effect of the robot.

#### 3.2.1. Forelimb Pitch Angle Adjustment Experiment

**Figure 13:**
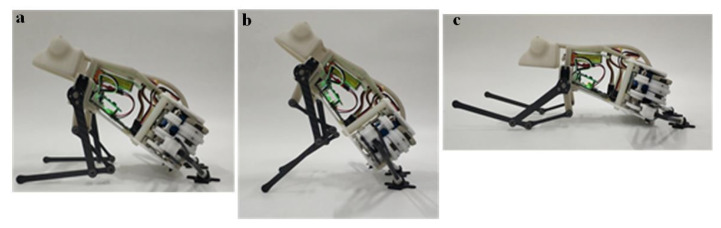
The pitch angle is adjusted by the forelimbs: (**a**): Minimum pitch angles of the prototype during takeoff; (**b**): Maximum pitch angles of the prototype during takeoff; (**c**) The large forward swing of the forelimb.

The forelimbs are mainly used to adjust the pitch angle of the robot and the cushioning and balance of the landing, so the test experiments of these two functions are mainly carried out. The pitch angle of the prototype can be adjusted by controlling the forelimbs through the upper computer to perform the two actions of “lifting up” and “pitching down”. Since the swing angle of the steering gear is not linearly related to the pitch angle, the operator needs to make several large-step coarse adjustments and small-step fine-tuning. Through the test, the maximum and minimum pitch angles of the prototype are shown in Figure 13a,b. The large forward swing of the forelimb is mainly used for the preparation of the robot for landing after jumping. This action needs to complete the entire action in the time period from the jump trigger to the landing; that is, there is a high requirement for the rapidity of the swing. Its complete motion feasibility and rapidity were tested, as shown in Figure 13c. The results are shown in Table 5. From the data in the table, it can be seen that the longest time it takes for the maximum pitch angle of 75° to reach the limit position of the forward swing is 0.19 s; that is, the rapidity of the forward swing action meets the requirements of jumping and landing.

#### 3.2.2. Hindlimb Movement Experiment

**Figure 14:**
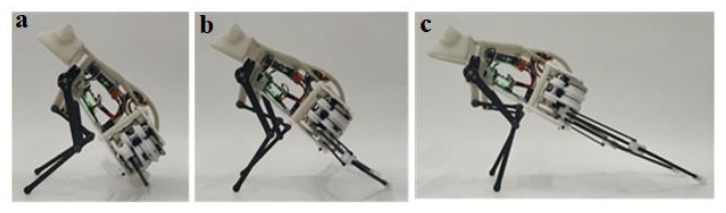
The movements of the hindlimb jumping: (**a**): Complete contraction of hindlimb; (**b**): Semi-extension of hindlimb; (**c**) Fully extension of hindlimb.

The hindlimb is the core of the jumping motion of the robot. During the test experiment, the completeness of the movements of this set of programs in a complete cycle is first tested. In order to adjust the energy released when the robot jumps, it is also necessary to test the power adjustment function. The upper computer sends separate instructions for each action to test, and the test results are shown in Figure 14. It can be seen that the hind limbs are able to complete all movements.

**Figure 15:**
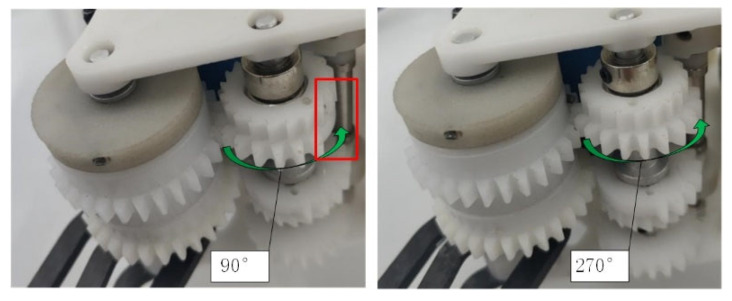
Schematic diagram of different torsion degrees of torsion spring (**left**) 90° (**right**) 270°.

The elastic potential energy is controlled by adjusting the torsion angle of the torsion spring by the host computer. Observe the relationship between the actual torsion angle and the set value after sending the charge command. The variation in the torsion angle of the torsion spring is shown in Figure 15. On the left, the incomplete gear is rotated 90 degrees, and the missing tooth part of the incomplete gear can be seen in the red box. On the right, the incomplete gear rotates 270 degrees. The missing tooth part of the incomplete gear has been turned to the back. It can be seen that the regulating function of the torsion spring works fine.

#### 3.2.3. Jump Experiment

**Figure 16:**
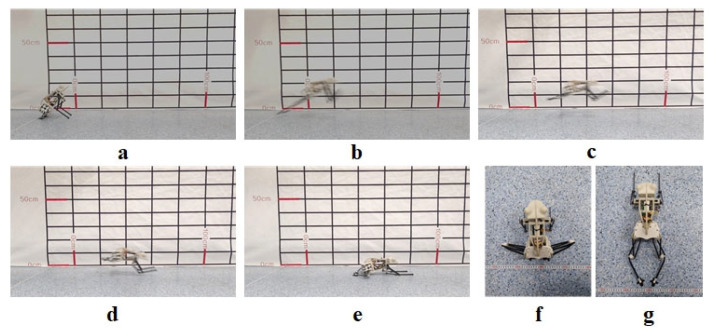
Prototype complete jump process: (**a**): Takeoff preparation; (**b**): Take-off phase; (**c**): Flight phase; (**d**): Landing phase; (**e**) Completely landing; (**f**): Top view before the jump; (**g**): Top view of the limb extension state.

Firstly, check whether the robot’s forelimbs and hindlimbs are functional. Figure 16f shows the limb contraction state, and Figure 16g shows the limb extension state. Then the jumping experiment is carried out. The complete jumping action of the robot is shown in Figure 16a–e. The jumping experiment result shows that the whole jumping process is consistent with the simulation result. The mechanism designed can realize the jump of the biomimetic robotic frog completely.

The torsion stroke of the torsion spring is set to the maximum, and the height and distance of the jump are changed by changing the pitch angle when the robot takes off. After many tests, the relationship between the pitch angle and the height and distance of the jump is shown in Table 6.

It can be roughly inferred from the table that when the take-off pitch angle of the robot is around 55°, the robot jumps the farthest.

The take-off pitch angle of the fixed robot is 55°, and the height and distance of the jump are adjusted by changing the accumulating stroke of the torsion spring. After many tests, the relationship between the spring torsion angle and the height and distance of the jump is shown in Table 7.

This experiment verifies the feasibility of adjusting the jump performance scheme by adjusting the accumulating stroke of the torsion spring.

### 3.3. Discussion

In the experiment, it was found that the energy output efficiency of the mechanism is not high, and there is a certain gap between the jump distance and jump height of the robot and the simulation results. Although the prototype can complete the jump, there are some disadvantages in the prototype. There are too many parts for the energy storage transmission structure, resulting in too much mass. The release efficiency of elastic energy of torsion spring is not high due to the influence of its own damping. The complex parts make the function of continuous jumping more unstable. After multiple jumps, the errors of structural parts are accumulated, and the control accuracy is decreased.

The hindlimb linkage structure is not yet the optimal size for output efficiency. The current structure of the hindlimb is optimized only for the overall dimension and the end trajectory. In future research, the relationship between the end force and the driving force of the nonlinear torsion spring will be analyzed to improve the output energy of the jump and optimize the structural parameters of the hindlimb.

## 4. Conclusions

In this paper, a novel jumping mechanism of a biomimetic robotic frog is proposed. Firstly, the structure and jumping characteristics of natural frogs are studied and analyzed in order to design the limbs of the biomimetic robotic frog. Based on the single-degree-of-freedom six-bar linkage, the design optimization of the structural parameters of the biomimetic frog hindlimb is given. Simplified forelimbs were designed based on the jumping function of the robot. Then a simplified model of a biomimetic robotic frog was established to simulate the jumping motion. Secondly, elastic energy storage and trigger mechanism were designed, including incomplete gears, one-way bearings, torsion springs, and so on, to realize the complete jumping function of the robot, that is, elastic energy storage and adjustment, elastic energy release, and rapid leg retraction. Thirdly, the experimental prototype of the biomimetic robotic frog was fabricated. According to the actual work requirements of the robot, the corresponding hardware and control system were built, and the experimental prototype was assembled to perform jumping experiments, which verified the rationality and feasibility of the mechanism design. It also provides a technical and theoretical basis for the development of an amphibious frog-inspired robot designed to adapt to complex land and water environments.

## 5. Patents

There are two patents resulting from the work reported in this manuscript:

1. Jizhuang Fan, et al. A frog-inspired amphibious robot and its motion control method, China. Patent number: ZL202110261064.7
2. Jizhuang Fan, et al. An elastic energy storage release mechanism and a control method China. Patent number: ZL202110261076.X

## Figures and Tables

**Table 1:** Link pivot coordinate optimization results.

	A	B	C	D	E	F	G	P_0_
X	0	−15.3453	7.3661	−8.8027	105.1479	84.2049	86.6153	0
Y	0	−7.0159	−2.4307	−0.0460	15.5495	17.4201	8.9830	−53.3969

**Table 2:** Performance parameter table of DS_BN22.

Voltage	No-Load Speed	Rated Torque	Locked-Rotor Torque	locked-Rotor Current	Weight
12 V	125 RPM	0.5 Nm	1.2 Nm	10 A	70 g

**Table 3:** Performance parameter table of SCS0009.

Voltage	Weight	No-Load Speed	Rated Torque
4.8~8 V	12.5 ± 0.2 g	143 RPM	7.6 kg·cm

**Table 4:** Performance parameter table of processing materials.

Material	Yield Strength/MPa	Density/g*mm-3	Intensity Density Ratio
45 # steel	355	7850	0.0452
40Cr	785	7850	0.1
6061 aluminum	55.2	2700	0.0204
7075-T6 aluminum	455	2810	0.1619
Titanium alloy	380	4510	0.0843
POM	70	1390	0.0504
Carbon Fiber	1320(0°), 61(90°)	1500	0.88(0°),0.0406(90°)

**Table 5:** Swing time from different pitch angles to the limit position.

Pitch Angle/°	35	45	55	65	75
Time/s	0.12	0.15	0.17	0.18	0.19

**Table 6:** Relationship between pitch angle and jump height and distance.

Pitch Angle /°	35	45	55	65	75
**height /m**	0.10	0.13	0.15	0.18	0.25
**distance /m**	0.32	0.41	0.50	0.44	0.34

**Table 7:** Relationship between torsion angle of spring and jump height and distance.

Torsion Angle /°	150	180	210	240	270
**height /m**	0.02	0.05	0.08	0.12	0.15
**distance /m**	0.08	0.13	0.30	0.42	0.50

## Data Availability

The datasets generated during and/or analyzed during the current study are available from the corresponding author upon reasonable request.
